# Succinate Enhances Lipolysis and Decreases Adipocytes Size in Both Subcutaneous and Visceral Adipose Tissue from High-Fat-Diet-Fed Obese Mice

**DOI:** 10.3390/foods12234285

**Published:** 2023-11-28

**Authors:** Tengteng Ji, Bing Fang, Ming Zhang, Yaqiong Liu

**Affiliations:** 1 Key Laboratory of Precision Nutrition and Food Quality, Department of Nutrition and Health, China Agricultural University, Beijing 100083, China; 2 School of Food Science and Chemical Engineering, Beijing Technology and Business University, Beijing 100048, China

**Keywords:** succinate, deposition, lipolysis, lipogenesis, thermogenesis

## Abstract

Obesity is a risk factor for many chronic diseases related to the overexpansion of adipose tissue during obesity, leading to metabolic dysfunction and ectopic lipids. Previous studies reported a close relationship between succinate and obesity and its co-morbidities, and studies have also reported on its anti-obesity potential. To confirm its efficacy in obesity interventions, we supplemented mice with obesity induced by a high-fat diet with succinate (1.5% *m*/*v* in drinking water) for 11 weeks without changing the diet. After succinate supplementation, the changes in body weight, adipose tissue deposition, glucose tolerance, energy expenditure and lipid metabolism were evaluated. It was found that succinate supplementation significantly decreased subcutaneous adipose tissue (HFD: 4239.3 ± 211.2 mg; HFD-SA: 3268.9 ± 265.7 mg. *p* < 0.05), triglyceride contents (decreased by 1.53 mmol/g and 0.39 mmol/g in eWAT and ingWAT, respectively, *p* < 0.05) and NEFA (decreased by 1.41 μmol/g and 1.31 μmol/g in eWAT and ingWAT, respectively, *p* < 0.05). The adipocytes’ sizes all significantly decreased in both subcutaneous and visceral adipose tissue (the proportion of adipocytes with diameters larger than 100 μm in eWAT and ingWAT decreased by 16.83% and 11.96%, respectively. *p* < 0.05). Succinate significantly enhanced lipolysis in adipose tissue (eWAT: *Adrb3*, *Hsl* and *Plin1*; ingWAT: *Hsl* and *CPT1a*; *p* < 0.05), whereas the expression of lipogenesis-related genes remained unchanged (*p* > 0.05). Succinate supplementation also enhanced the activity of BAT by stimulating the expression of *Ucp1* and *Cidea* (*p* < 0.05). Our results reported that succinate has a potential beneficial effect on obesity pathogenesis but cannot efficiently decrease bodyweight.

## 1. Introduction

At present, obesity is an urgent problem to be addressed [1] that is associated with an increase in the morbidity and mortality of several diseases, especially cardio-vascular diseases and diabetes [2,3]. Obese populations are characterized by a significant increase in the mass of both subcutaneous and visceral adipose tissues, accompanied by an expansion of adipose tissue, including changes in the adipocytes’ number and/or size which varies with energy status [4]. At the same time, excessive lipid accumulation triggers a redistribution of lipids among metabolic organs, thus altering lipid metabolism [5] and leading to many of the diseases mentioned above. Therefore, plenty of studies are focused on investigating obesity pathogenesis and useful treatments to prevent obesity.

Succinate, as an intercalator in the tricarboxylic acid (TCA) cycle, has been reported to bind to the SUCNR1 receptor and make the difference in the anti-inflammatory response of macrophages [6], which are involved in the obesity pathogenesis [7]. Succinate was found to improve glucose homeostasis and metabolism in animal studies via inducing adipocyte browning or a substrate of intestinal gluconeogenesis, thereby ameliorating obese or diabetic pathologies [8,9,10,11,12,13,14,15]. In addition, it has been suggested that the buildup of succinate in hypoxic adipose tissues acts as a metabolic signature connecting endoplasmic reticulum pressure, inflammation, and cAMP/PKA activation, which facilitate lipolysis [16]. In addition, epidemiological data have shown that succinate may prevent chronic inflammation by regulating the UCP1 expression in preadipocytes [17]. However, there is almost no immediate proof for the anti-obesity potential of succinate, resulting in the current lack of conclusive evidence for its role in preventing mouse obesity, and the available data on the weight-loss effects of succinate are derived from a short-term high-fat diet (HFD) of approximately 3–4 weeks duration [8,9,11,12,13,14,15]. Moreover, when investigating the relevance of succinate concentrations to the metabolic disease, several papers have described increased levels of circulating succinate in both obese and/or diabetic patients [6,18,19] and rodents [20,21,22,23]. In addition, improved glycemic control and inflammation in obese mice were associated with the consumption of cyclic succinate [17,24]. Studies have also reported an unchanged level of succinate in humans with metabolic dysfunction [25,26], leading to uncertainty in the application of succinate in anti-obesity therapies. Therefore, we conducted this experimentation to evaluate the impact of succinate on obesity pathogenesis evoked by a long-term HFD.

Here, we studied the effects of succinate on adipose deposition and metabolic dysfunction associated with obesity in obese mice. As a food additive and dietary supplement, the mice were kept on an HFD for the entire duration of the experiment to exclude the effect of decreased calorie intake due to diet changes. Our study will give direct proof for the efficiency of succinate supplementation in anti-obesity therapy.

## 2. Materials and Methods

### 2.1. Animals and Experimental Design

**Figure 1:**
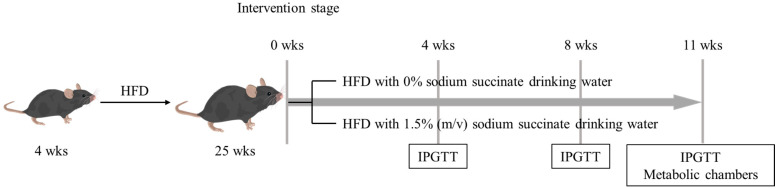
**The experimental flow graph.** Twenty-five-week-old obese C57BL/6J mice (high-fat-diet-induced from 4 weeks to 25 weeks) were supplemented with 0 or 1.5% (*m*/*v*) sodium succinate for 11 weeks. During the experimental period, IPGTT was performed in the fourth and eighth weeks. At the end of the experiment (11 weeks), IPGTT and metabolic chambers were performed.

Our animal protocols were reviewed and approved by the Animal Care and Ethics Committee of the China Agricultural University (AW61212202-5-1). Three-week-old male C57BL/6J mice were purchased from SPF (Beijing, China) Biotechnology Co., Ltd. and acclimatized for 1 week. The animals were housed individually. Beginning from 4 weeks old, the mice were kept on an HFD (60% energy from fat, D12492, Research Diets, Beijing, China) for 21 weeks, the percentage of energy for each macronutrient of which is listed in Table 1. Then, we divided the mice into two groups: HFD only (HFD group, *n* = 6) and HFD combined with sodium succinate (224731-500G, Sigma-Aldrich, Beijing, China) (HFD-SA group, *n =* 7) for 11 weeks. The experimental flow graph is shown in Figure 1. The drinking water with addition of 1.5% succinate (*m*/*v*) was freshly prepared and changed every day (the dose and duration were based on the study by Mills et al. [8]). We placed the mice in a chamber with controllable (23 °C) temperatures and a photoperiod cycle of 12/12 h. Additionally, we recorded weight gain, food intake and water intake weekly. At the termination of treatment, mice were euthanized via the inhalation of CO_2_ and cervical dislocation. Blood samples, epididymal (eWAT), perirenal (pWAT), inguinal (ingWAT) and axilla (aWAT) white adipose tissues, brown adipose tissue (BAT) and livers were rapidly collected from individual mice.

### 2.2. Intraperitoneal Glucose Tolerance Test (IPGTT)

After fasting for 14 h, the mice were intraperitoneally injected with 1 g/kg glucose solution (10 mg/mL). Blood glucose at time points of 0, 15, 30, 60, 90, 120, and 180 min was measured from blood in the tail using a blood glucose meter (Roche, ACCU-CHEK Active, Penzberg, Germany). The region below the baseline of the IPGTT curve was computed following the methodology reported by Virtue et al. [27].

### 2.3. Metabolic Phenotyping

The total body energy metabolism of the mice was analyzed using a metabolic chamber (Panlab, Oxylet Pro, Panlab, Barcelona, Spain). Each mouse was fed separately and acclimated 48 h in advance to minimize the effects of stress on the metabolic phenotypes. During this time, the room was programmed for a photoperiod cycle of 12/12 h. The instrument continuously collected data every 5 min for 48 h. The following parameters were monitored: oxygen consumption (VO_2_), carbon dioxide production (VCO_2_), respiratory quotient (RQ) and energy expenditure (EE) [28]. Subsequent data processing was performed according to the protocols of Matthias et al. [29]. We performed a covariate analysis of the primary data. This was normalized to lean mass to express the gas exchange parameters.

### 2.4. Body Temperature and Cold Exposure

Mice were maintained individually in pre-chilled cages and set to a low temperature (4 °C) environment for 6 h with unrestricted availability of food and water [30]. Body temperature was taken individually using an IR camera (FLIR T420, FLIR Systems AB, Täby, Sweden).

### 2.5. Histologic Analysis

The organizations were immobilized in 4% paraformaldehyde at an ambient temperature and then embedded in paraffin, sliced and placed on slides. Organization slices (3 μm) were dewaxed, rehydrated, stained by hematoxylin and eosin (Eosin Y Stain Solution, Solarbio, G1100, Beijing, China; Mayer’s Hematoxylin Stain Solution, G1080, Beijing, China). Three images per section, two sections per mouse and three mice in each group were analyzed. The digital images were collected using an upright microscope (Leica DM6 B) equipped with a 10× or 20× or 63 × objective lens. The adipocyte diameters were analyzed by Image-Pro Plus software 6.0 (Media Cybernetics, Rockville, MD, USA) [31].

### 2.6. Oil Red O Staining 

Lipid buildup in the liver was determined by Oil Red O dyeing. Frozen sections (10 μm) of liver tissues were immobilized with 4% paraformaldehyde and washed with water. Then, we soaked the slides in 60% isopropyl alcohol and stained in Oil Red O (saturated Oil Red O solution diluted 3:2 with distilled water, Solarbio, G1260, Beijing, China) at an ambient temperature for 10 min. The sections were cleaned twice in 60% isopropanol and colored with Mayer’s hematoxylin stain solution (Solarbio, G1080, Beijing, China) for 30 s. We applied Image-Pro Plus software 6.0 (Media Cybernetics, USA) to count the mathematical statistics of the Oil Red O images and calculated the area of lipid that was dyed red [32].

### 2.7. Quantitative Real-Time qPCR

Total RNA was extracted from eWAT, ingWAT, BAT and liver tissues using TRIzol (Invitrogen, Carlsbad, CA, USA), which was then back-transcribed to cDNA using the All-In-One 5X RT Mastermix (Abm, G492, Beijing, China). A total of 1500 ng total RNA was used. We analyzed gene expression using real-time qPCR with SYBR Green (Takara, RR82LR, Beijing, China) and normalized to GAPDH. The primer pair sequences were as follows: *ACC*: forward, 5′-GATGAACCATCTCCGTTGGC-3′; reverse, 5′-CCCAATTATGAATCGGGAGTGC-3′; *Adrb3*: forward, 5′-GGCCCTCTCTAGTTCCCAG-3′; reverse, 5′-TAGCCATCAAACCTGTTGAGC-3′; *Atgl*: forward, 5′-CTGAGAATCACCATTCCCACATC-3′; reverse, 5′-CACAGCATGTAAGGGGGAGA-3′; *Cebpa*: forward, 5′-CAAGAACAGCAACGAGTACCG-3′; reverse, 5′-GTCACTGGTCAACTCC AGCAC-3′; *Cidea*: forward, 5′-TGCTCTTCTGTATCGCCCAGT-3′; reverse, 5′-GCCGTGTTAAGG AATCTGCTG-3′; *CPT1a*: forward, 5′-CACTGCAGCTCGCACATTAC-3′; reverse, 5′-CCAGCACAAAGTTGCAGGAC-3′; *Dgat2*: forward, 5′-GCGCTACTTCCGAGACTACTT-3′; reverse, 5′-GGG CCTTATGCCAGGAAACT-3′; *GAPDH*: forward, 5′-TGTGTCCGTCGTGGATCTGA-3′; reverse, 5′-CCTGCTTCACCACCTTCTTGA-3′; *Hsl*: forward, 5′-TCCTCAGAGACCTCCGACTG-3′; reverse, 5′-ACACACTCCTGCGCATAGAC-3′; *Plin1*: forward, 5′-CAAGCACCTCTGACAAGGTT C-3′; reverse, 5′-GTTGGCGGCATATTCTGCTG-3′; *Pparγ*: forward, 5′-GGAAGACCACTCGCATT CCTT-3′; reverse, 5′-TCGCACTTTGGTATTCTTGGAG-3′, *UCP1*: forward, 5′-GCTTTGCCTCAC TCAGGATTGG-3′; reverse, 5′-CCAATGAACACTGCCACACCTC-3′.

### 2.8. Biochemical Analysis for Liver, Adipose Tissue and Serum Samples

The triglyceride and non-esterified fatty acids (NEFAs) contents in eWAT, ingWAT and liver were determined using colorimetric kits (Nanjing Jiancheng Bioengineering Institute, A110-1-1, Beijing, China; Boxbio, AKFA008M, Beijing, China). The insulin level in serum was measured using the Ultra-Sensitive Mouse Insulin ELISA Kit (Crystal Chem USA, 90080, Beijing, China).

### 2.9. Statistical

We expressed the data as the mean ± s.e.m. Student’s *t*-test was used for pairwise comparisons of *p* values. One-way ANOVA for multiple comparison of variables was used for Tukey’s multiple comparison tests. Significance was marked as a *p*-value < 0.05 (labeled with *) or 0.01 (labeled with **). Biological replication was used in this experiment. Additionally, the mice were randomly grouped.

## 3. Results

### 3.1. Succinate Improved Glucose Intolerance, Adipose Deposition and Energy Expenditure in Obese Mice

**Figure 2:**
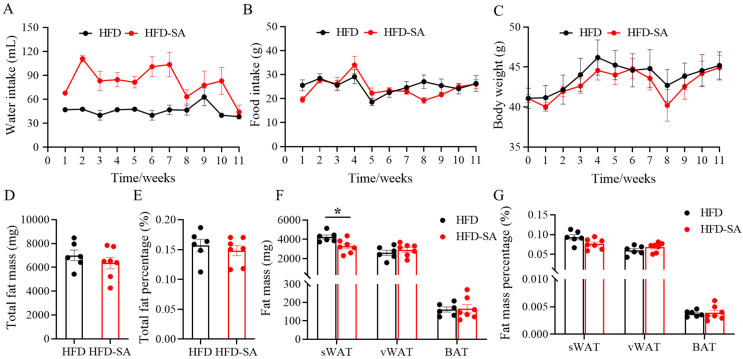
**Succinate decreased subcutaneous adipose tissue (sWAT) deposition.** (**A**) Water intake; (**B**) food intake; (**C**) body weight; (**D**) total fat mass; (**E**) total fat percentage; (**F**) weight and (**G**) percentage of sWAT, vWAT and BAT of mice in HFD and HFD-SA groups (HFD: *n =* 6; HFD-SA: *n =* 7). Values are expressed as mean ± s.e.m. of biologically independent samples. *p*-values were determined using unpaired *t*-tests. * *p* < 0.05.

During the whole experiment, mice showed no aversion to water containing sodium succinate (Figure 2A), although water intake in the HFD-SA was actually twice as much as that in the HFD. This may be due to the fact that the use of succinate as a food additive can enhance the umami flavor and increase water intake. There were also no noticeable changes in food consumption between the two groups (Figure 2B, *p* > 0.05). The addition of succinate into the drinking water for 11 weeks did not prevent the effects of an HFD, with no apparent discrepancy being recorded in the body weight (Figure 2C), total fat mass (Figure 2D) or the total fat percentage (Figure 2E). When the deposition of adipose tissue was evaluated, it was found that the weight and percentage of subcutaneous adipose tissue (sWAT) decreased (*p* < 0.05 in Figure 2F and *p* = 0.059 in Figure 2G), whereas visceral adipose tissue remained unchanged (vWAT, *p* > 0.05).

**Figure 3:**
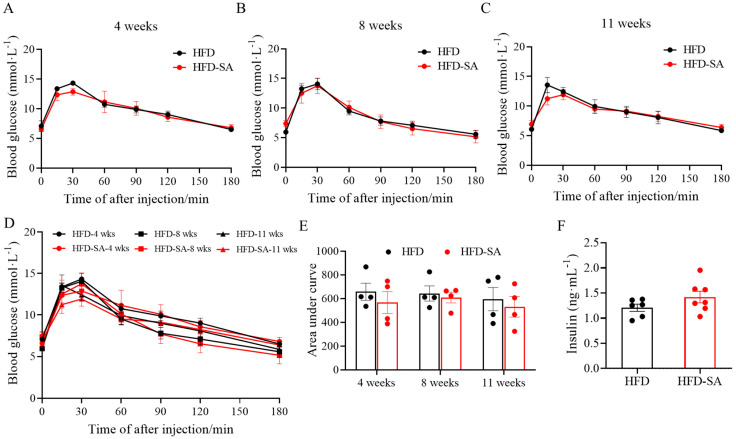
**Effect of succinate supplementation on glucose tolerance.** (**A**–**C**) IPGTT graphs after 4 weeks (**A**), 8 weeks (**B**) and 11 weeks (**C**) of intervention with succinate; (**D**) the mixed IPGTT graphs and (**E**) area under the baseline curve in the IPGTT graph (*n =* 4); (**F**) serum contents of insulin after the 11-week intervention (HFD: *n =* 6; HFD-SA: *n =* 7). Values are expressed as mean ± s.e.m. of biologically independent samples. *p*-values were determined using unpaired *t*-tests.

**Figure 4:**
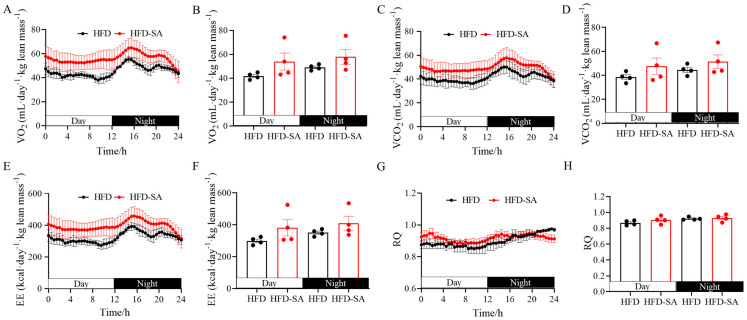
**Effect of succinate supplementation on energy expenditure.** (**A**) Oxygen consumption (VO_2_) over 24 h and the (**B**) quantitative mean values during the day and nighttime; (**C**) carbon dioxide production (VCO_2_) over 24 h and the (**D**) quantitative mean values during the day and nighttime; (**E**) whole-body energy expenditure (EE) over 24 h and the (**F**) quantitative mean values during the day and nighttime; (**G**) respiratory quotient over 24 h and the (**H**) quantitative mean values during the day and nighttime, which is defined as the ratio of carbon dioxide production and oxygen consumption at the same time (*n =* 4). Values are expressed as mean ± s.e.m. of biologically independent samples. *p*-values were determined using unpaired *t*-tests.

As for the glucose metabolism, it can be seen from Figure 3 that succinate did not improve the glucose intolerance induced by HFD (Figure 3A–E, *p* > 0.05), although the circulating insulin levels increased slightly in the HFD-SA group in the eleventh week (Figure 3F, *p* > 0.05). Furthermore, succinate supplementation also slightly increased the VO_2_ (Figure 4A,B, *p* > 0.05), VCO_2_ (Figure 4C,D, *p* > 0.05) and energy expenditure (Figure 4E,F, *p* > 0.05) of the obese mice in both the light and night phases. In addition, RQ was not changed between the two groups (Figure 4G,H).

### 3.2. Succinate Decreases Triglyceride Content in Adipose Tissue and Adipocyte Size

**Figure 5:**
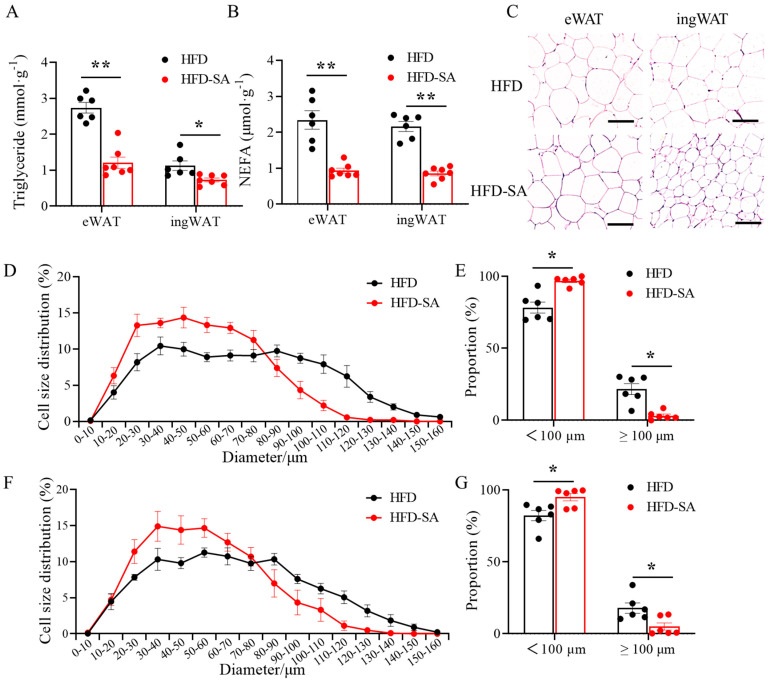
**Succinate inhibited HFD-induced lipid accumulation.** (**A**) Triglyceride and (**B**) NEFA contents in eWAT and ingWAT (HFD: *n =* 6; HFD-SA: *n =* 7). (**C**) Representative images of hematoxylin and eosin (H&E) staining of eWAT and ingWAT (20× magnification; scale bars, 0.1 mm). (**D**) The distribution of adipocytes diameters in eWAT. Adipocyte size was measured using Image-Pro Plus software, and we chose six fields for each mouse and counted 50–80 cells for each field. (**E**) Proportion of adipocytes with diameters greater than 100 μm in eWAT. (**F**) The distribution of adipocytes diameters in ingWAT. Adipocyte size was measured using Image-Pro Plus software, and we chose six fields for each mouse and counted 50–80 cells for each field. (**G**) Proportion of adipocytes with diameters greater than 100 μm in ingWAT (*n =* 6). Values are expressed as mean ± s.e.m. of biologically independent samples. *p*-values were determined using unpaired *t*-tests. * *p* < 0.05, ** *p* < 0.01.

After supplementation with succinate, the contents of triglyceride and NEFA in eWAT and ingWAT both significantly decreased (Figure 5A,B, *p* < 0.05). In addition, adipocyte sizes in eWAT and ingWAT also significantly decreased (Figure 5C–G, *p* < 0.05), and the proportion of adipocytes with diameters larger than 100 μm in eWAT and ingWAT decreased from 20% and 17% to 3.17% and 5.04%, respectively (Figure 5E,G, *p* < 0.05).

### 3.3. Succinate Enhances Lipolysis in Both Adipose Tissue and Liver

**Figure 6:**
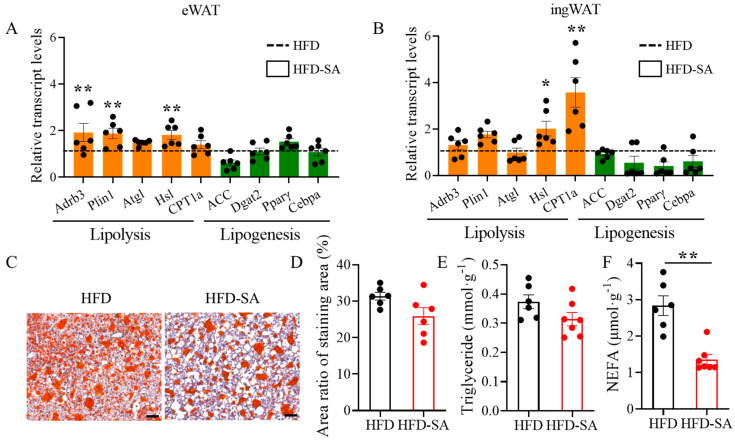
**Succinate enhances lipolysis both in adipose tissue and liver.** (**A**,**B**) Relative mRNA expression of genes involved in lipolysis (orange, including *Adrb3*, *Plin1*, *Atgl*, *Hsl*, *CPT1a*) and lipogenesis (green, including *ACC*, *Dgat2*, *Pparγ*, *Cebpa*) in (**A**) eWAT and (**B**) ingWAT measured using quantitative real-time PCR (*n =* 6). (**C**) Representative images of Oil-Red-O-stained liver sections (10× magnification; scale bars, 0.1 mm, *n =* 6). (**D**) Area ratio of Oil Red O staining area was measured using Image-Pro Plus software (*n =* 6). Liver tissues were analyzed for (**E**) triglyceride and (**F**) NEFA contents (HFD: *n =* 6; HFD-SA: *n =* 7). Data are expressed as mean ± s.e.m. of biologically independent samples. *p*-values were determined using unpaired *t*-tests. * *p* < 0.05, ** *p* < 0.01.

Succinate supplementation significantly increased the mRNA expression of *Adrb3*, *Hsl* and *Plin1* in eWAT (Figure 6A, *p* < 0.05) and *Hsl* and *CPT1a* in ingWAT (Figure 6B, *p* < 0.05), which are all involved in lipolysis. However, the mRNA expressions of the lipogenesis-related genes, including *Pparγ*, *Cebpa*, *ACC* and *Dgat2*, in both eWAT and ingWAT, were unchanged (Figure 6A,B, *p* > 0.05). 

Obesity induces ectopic fat deposition in the liver. Through Oil Red O staining, succinate supplementation was found to decrease lipid deposition by 17.5% (Figure 6C,D, *p* > 0.05) and triglyceride contents in the liver slightly decreased (Figure 6E, *p* > 0.05). The contents of NEFA in the liver declined significantly (Figure 6F, *p* < 0.05).

### 3.4. Succinate Enhances the Activation of BAT Thermogenesis

**Figure 7:**
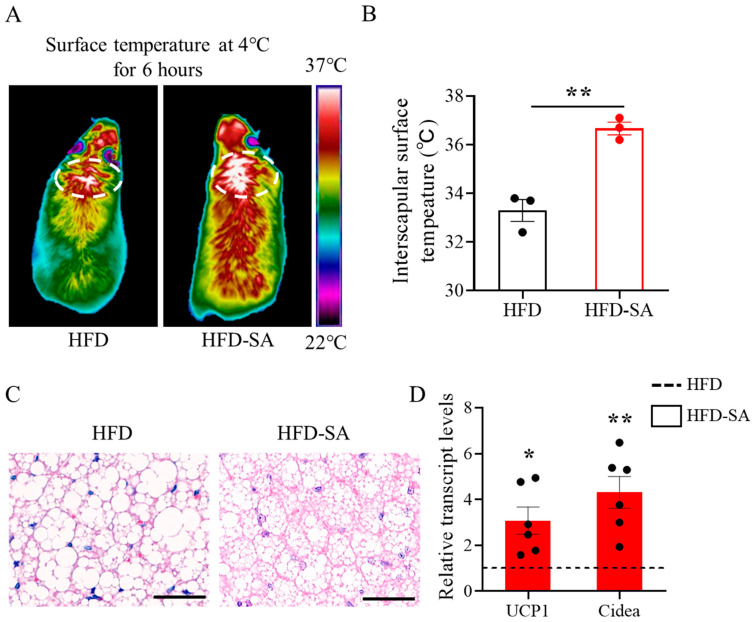
**Succinate enhances the activation of BAT thermogenesis in mice fed with an HFD.** (**A**) Representative infrared thermography of HFD and HFD-SA mice exposed to 4 °C for 6 h. (**B**) Surface temperature was quantified in the interscapular BAT area indicated by circle (*n =* 3). (**C**) Representative images of hematoxylin and eosin (H&E) staining of BAT (63× magnification; scale bars, 0.05 mm). (**D**) Relative mRNA expression of genes (including UCP1 and Cidea) in BAT measured using quantitative real-time PCR (*n =* 6). Data are expressed as mean ± s.e.m. of biologically independent samples. *p*-values were determined using unpaired *t*-tests. * *p* < 0.05, ** *p* < 0.01.

Thermographic imaging following cold exposure in mice revealed a significant increase in interscapular temperature in mice supplemented with succinate (Figure 7A,B, *p* < 0.05). Additionally, H&E staining revealed that the BAT after succinate supplementation exhibited a denser structure (Figure 7C). Consistent with the increased surface temperature, the mRNA expressions of *UCP1* and *Cidea* in the BAT of mice in the HFD-SA group increased significantly (Figure 7D, *UCP1*: *p* < 0.05; *Cidea*: *p* < 0.01), which is related to thermogenesis.

## 4. Discussion

Adipose tissue, a dynamic organ distributed around the whole body, has the infinite capacity of overexpansion during obesity [33]. Our findings suggested that succinate intervention slowed the expansion of adipose tissue in HFD-induced obesity. After an 11-week succinate intervention, the total adipose tissue weight of the obese mice decreased (Figure 2D, *p* > 0.05), especially the sWAT mass (Figure 2F, *p* < 0.05). This effect was mainly due to the decrease in triglyceride and NEFA contents in adipocytes in eWAT and ingWAT (Figure 5A,B, *p* < 0.05), and partly related to the slight increase in energy consumption (Figure 4F, *p* > 0.05). Triglycerides are predominantly stockpiled in adipocytes as lipid droplets, which continue to grow up to 100 μm in diameter and can induce chronic inflammation with further growth [34]; therefore, the decrease in the proportion of adipocytes to below 100 μm may alleviate obesity pathogenesis. Moreover, in obese individuals, the basal lipolysis of hypertrophic adipocytes is elevated, resulting in the leakage of NEFA [35]. Large amounts of NEFA are absorbed by the liver or muscle, leading to ectopic lipid profusion [36]. In the liver after the succinate intervention, the content of NEFA was significantly reduced (Figure 6F, *p* < 0.01), indicating that succinate can reduce the deposition of hepatic ectopic fat in high-fat-diet-induced obesity in mice to some extent.

Adipocyte size is regulated by lipolysis and lipogenesis. The lipolysis process is mainly catalyzed by lipolytic enzymes, including the rate-determining adipose triglyceride lipase (ATGL) and hormone-sensitive lipase (HSL) [37]. Meanwhile, perilipin 1 (Plin1), a protein situated on the surface of lipid droplets in adipocytes, helps lipase enter lipid droplets [38]. Notably, the qPCR results showed that both eWAT and ingWAT showed an increased transcription of HSL and Plin1 (Figure 6A,B, *p* < 0.05). However, the lipogenesis process is mainly catalyzed by the peroxisome proliferator-activated receptor γ (PPARγ), CCAAT enhancer-binding protein alpha (C/EBPa), diacylglycerol-O-acyltransferase 2 (DGAT2) and the rate-limiting enzyme acetyl-coenzyme A carboxylase alpha (ACC) [39,40]. The expression of these genes after succinate supplementation did not significantly change, although the declining trend in ingWAT was more obvious than that in eWAT (Figure 6A,B, *p* > 0.05). This can explain the fat pad mass results, in which sWAT showed a more significant weight loss than vWAT (Figure 2F, *p* < 0.05). Meanwhile, the results of Serena et al. [18] showed that sWAT is more sensitive to succinate than vWAT, which is also in line with our findings. Overall, the current evidence suggests that succinate can influence lipolysis and lipogenesis, and thus reduce lipid deposition, through modulation of a range of different enzymes and proteins.

For obesity, the excessive intake of energy is the main cause of occurrence, whereas BAT, as a type of adipose tissue that is specifically used in energy consumption, plays a central part in maintaining energy balance [41]. Additionally, the literature has increasingly shown that stimulating BAT thermogenesis has become a critical approach to combat obesity [42,43,44,45,46]. In our study, the results revealed that the succinate supplementation enhanced BAT activity, which is in agreement with the findings of Mills et al. [8]. In addition, thermogenesis through uncoupling protein 1 (UCP1) is a primary mechanism for BAT to increase energy expenditure [47]. Meanwhile, cell-death-inducing DNA fragmentation factor-like effector A (Cidea), as a lipid-droplet-related protein, can also regulate the energy expenditure [48]. Our results suggested that succinate stimulated UCP1 and Cidea expression (Figure 7D, *p* < 0.05), thereby enhancing the activity of BAT.

Several studies have focused on circulating succinate levels and their relationship with body health, reporting that a number of metabolic diseases could affect circulating succinate levels. Some data suggest elevated serum succinate circulating levels in obese mice, followed by a population-based experiment that found no change in serum succinate levels in diabetic patients compared to controls [26]. However, in a cohort of 91 subjects, plasma succinate levels were significantly increased in obese compared to in lean patients [18]. In another group of 2248 healthy, middle-aged adults from the United Kingdom, circulating succinate was inversely associated with total adipose tissue mass [49]. Given the uncertainty in the trend regarding succinate levels at the organism level, we performed further monitoring and analysis using metabolomics. As an intermediate product of the TCA cycle, we speculated that succinate may be metabolized and utilized by the organism, and our data suggest that the levels of succinate in either serum or in ingWAT did not change significantly after the intervention (Supplementary Figure S1B,D, *p* > 0.05), which is consistent with the results of some studies [18,25,50]. These results suggest that a metabolically impaired organism has a high level of succinate due to its inability to utilize it, and gut microbiota were reported to utilize succinate as a substrate for intestinal glucose metabolism [11]. In addition, other metabolites involved in glycolysis and the TCA cycle also did not significantly change, indicating that the supplementation of succinate did not affect the energy metabolism but enhanced lipolysis. This study directly evaluated the effect of succinate on the obese mice without changing the HFD, and although succinate only had a beneficial effect on the excessive hypertrophy of adipocytes through the stimulation of lipolysis, as well as the function of BAT, further studies could evaluate its effect on adipose tissue under a lean condition. In our experiment, only two groups were considered: an obese group and an obese group with succinate. A healthy control group was missing, which makes it difficult to ascertain how much of an impact the HFD and succinate treatments exerted. In addition, the heterogeneity of different fat pools may lead to functional differences. They could also be used as potential targets for the treatment or alleviation of obesity-related metabolic disorders in the future.

## 5. Conclusions

Our data show that dietary succinate supplementation could efficiently decrease adipose tissue mass deposited in the subcutis (inguinal adipose tissue) and the contents of triglycerides in the liver in diet-induced obese mice. The decrease in adipose tissue mass was due to the enhanced lipolysis and inhibited lipogenesis, which reduced adipocyte size in both ingWAT and eWAT; this may be the reason for the improved glucose tolerance. Our study evaluated long-term succinate supplementation under the obese condition and provided new ideas for future dietary interventions for metabolic diseases including obesity.

## Figures and Tables

**Table 1:** The percentage of energy for each macronutrient of the diet.

Diet	Catalog#	Cal Density (kcal/g)	Fat (%)	Protein (%)	Carbohydrate (%)
HFD	D12492	5.24	60	20	20

## Data Availability

The data used to support the findings of this study can be made available by the corresponding author upon request.

## Supplementary Materials

The following supporting information can be downloaded at: https://www.mdpi.com/article/10.3390/foods12234285/s1, Figure S1. Formulation metabolites contents involved in glycolysis and TCA cycle in serum and ingWAT. References [51,52] are cited in the Supplementary Materials. Targeted energy metabolomics of serum and ingWAT samples were measured using the LC-MS/MS method according to Liu [51] and Fu [52] et al.
