# Supplementary material for: Succinate Enhances Lipolysis and Decreases Adipocytes Size in Both Subcutaneous and Visceral Adipose Tissue from High-Fat-Diet-Fed Obese Mice
Source: Foods. 2023 Nov 28;12(23):4285. doi: 10.3390/foods12234285 (PMC10706267; doi:10.3390/foods12234285)
Supplement: Supplementary file 1 [file foods-12-04285-s001.zip › foods-2734381-supplementary.pdf]

Succinate enhances lipolysis and decreases adipocytes sizes both in the subcutaneous and visceral adipose tissue from high-fat diet fed obese mice

Tengteng Ji [1], Bing Fang [1,*], Ming Zhang [2] and Yaqiong Liu [1]

[1] Key Laboratory of Precision Nutrition and Food Quality, Department of Nutrition and Health, China Agricultural University, Beijing 100083, China
[2] School of Food Science and Chemical Engineering, Beijing Technology and Business University, Beijing 100048, China
* Correspondence: bingfang@cau.edu.cn; Tel./Fax: +86-10-62736344

Supplementary Figure S1

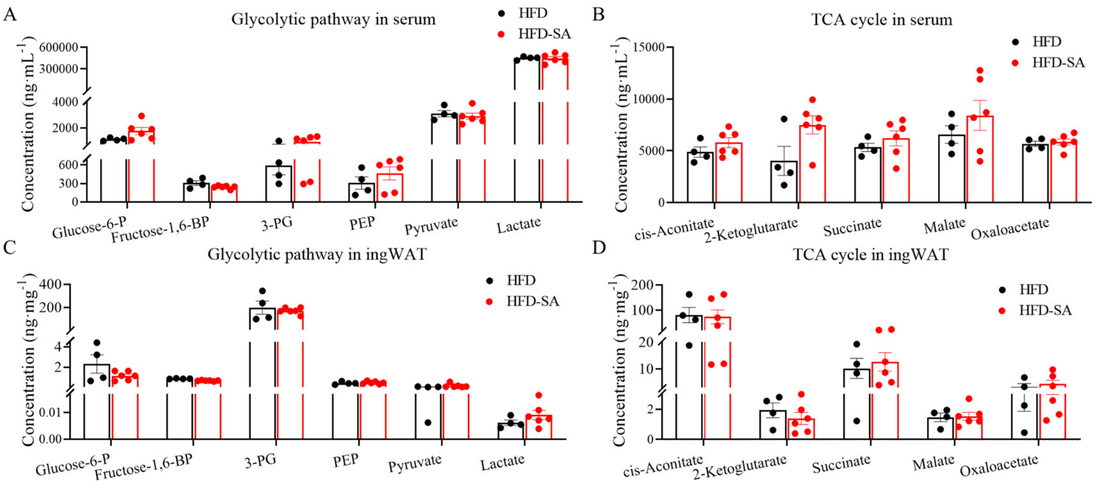

Supplementary Figure S1. The contents of metabolites involved in energy metabolism. The concentrations of glycolytic pathway metabolites (A) and Tricarboxylic acid (TCA) cycle (B) in serum after succinate supplementation. The concentrations of glycolytic pathway metabolites (C) and TCA cycle (D) in ingWAT after succinate supplementation (HFD: n=4; HFD-SA: n=6). Glycolytic pathway metabolites include Glucose-6-phosphate (Glucose-6-P), Fructose-1,6-bisphosphate (Fructose-1,6-BP), 3-phosphoglycerate (3-PG), Phosphoenolpyruvate (PEP), Pyruvate, Lactate. TCA cycle metabolites include cis-Aconitate, 2-Ketoglutarate, Succinate, Malate, Oxaloacetate. Quantitative data are mean ± s.e.m. of biologically independent samples. p-Values were determined by One-way ANOVA. *$p < 0.05$, **$p < 0.01$.
